# Impersonation and personification in mid-twentieth century mathematics

**DOI:** 10.1177/0073275320924571

**Published:** 2020-06-26

**Authors:** Michael J. Barany

**Affiliations:** University of Edinburgh, UK

**Keywords:** Authorship, mathematics, pseudonyms, fraud, integrity, abstracting

## Abstract

Pseudonymous mathematician Nicolas Bourbaki and his lesser-known counterpart E.S. Pondiczery, devised respectively in France and in Princeton in the mid-1930s, together index a pivotal moment in the history of modern mathematics, marked by international infrastructures and institutions that depended on mathematicians’ willingness to play along with mediated personifications. By pushing these norms and practices of personification to their farcical limits, Bourbaki’s and Pondiczery’s impersonators underscored the consensual social foundations of legitimate participation in a scientific community and the symmetric fictional character of both fraud and integrity in scientific authorship. To understand authorial identity and legitimacy, individual authors’ conduct and practices matter less than the collective interpersonal relations of authorial assertion and authentication that take place within disciplinary institutions.

## Introduction: A life in reverse

There is a general pattern to becoming a modern mathematical author. First comes a person who grapples with the discipline’s foundations, enters professional communities, finds collaborators, and gains wider recognition through publications followed by citations. With luck and wit, the author may travel the world and live on in the literature as an eponym or legend.

Nicolas Bourbaki’s story ran more or less in reverse. His surname, borrowed from a military personage, was in the air at the elite French École normale supérieur when, circa 1923, a student manifested it in an obscure accent from behind a false beard in a prank lecture as an eponym: Bourbaki’s theorem.^1^

Another student in the audience, André Weil, went on to obtain a doctorate in mathematics and found his first professional appointment in Aligarh, India.

There, Weil met the Harvard-educated Indian mathematician Damodar D. Kosambi, who, with Weil’s encouragement, worked the eponym into the title and discussion of his second ever mathematical paper.^2^ Kosambi gave Bourbaki a first initial (D), a nationality (Russian), a cause of death (“acute lead poisoning [i.e., gunshot] during the revolution”), and an unpublished *Nachlass* reputedly kept in the Leningrad Academy. In lieu of details of Bourbaki’s mathematics, he referred to a recent work then available only in Russian, thanking Weil for furnishing a reprint. A footnote anticipated both the (real) Russian paper’s and the (fictional) Bourbaki manuscripts’ hopeful translation and adjoined further details of Bourbaki’s death by way of a joke about the Cyrillic alphabet.^3^ The implication was that Kosambi’s countrymen could not tell a genuine obscure Russian mathematician and theorem from a fake, or were not worldly enough to care.

None of these biographical details outlived Kosambi’s article, but the name Bourbaki went on to adorn articles and textbooks that would fundamentally reshape the practices, ideologies, cultures, and politics of modern mathematics. Beginning in late-1934, Weil joined with a small band of fellow upstarts in an ambitious project of curricular and conceptual reform that would continue for decades hence.^4^ They adopted the collective pseudonym Bourbaki, christened Nicolas by Eveline de Possel, who would divorce Bourbaki cofounder René de Possel and marry Weil in 1937. Over the years, the critical and contrarian Bourbaki acquired a distinctive mathematical style and a fragmentary and sometimes-contradictory body of biographical lore, and became the only non-deceased figure included in the *Dictionary of Scientific Biography*.^5^

Because Bourbaki could not travel, write, or speak for himself, his personal manifestations relied upon impersonations. The self-styled “collaborators” of Bourbaki jointly prepared authoritative texts and joined with others to represent Bourbaki in a variety of contexts, often teasing or transgressing disciplinary norms as they did so. Relishing myth and misdirection, Bourbaki’s collaborators propounded his pseudonymous authorship as an open secret around which to establish or evade various forms of credit and responsibility in and beyond the mathematics profession. Their text-based personification reflected through parody how a widening world of mathematicians with strange names and biographies came to know and interact with each other, and dramatized the challenges and opportunities of an emerging global authorial order.^6^

The problems of trust and verification are endemic to modern science, with the social problem of knowing and trusting people crucially mediating the epistemic problem of knowing and trusting the facts of the world.^7^ Early modern conceptions of scientific authorship focused on assigning legal responsibility for textual claims rather than giving credit for the creative acts that underwrote them, and the tension between credit and responsibility defined competing economies of authorship over the ensuing centuries.^8^ Conceptions of fraud and integrity, in this tradition, have correspondingly focused on the relationships between individual named authors and associated facets of conduct and accountability, a focus often ill-matched to the structures and geographies of modern and contemporary research.^9^ Latter-day regimes of secrecy and bureaucratic control of authorship that subordinate individual labor under the name of a sacralized figurehead have been glossed as “the ‘Bourbakification’ of science,” in reference to the Bourbaki collective’s concerted obfuscation, the collaborative ideal this supported (in principle), and the havoc it made for attribution.^10^

Such subordination may have a special character for mathematical texts written with a demonstrative posture based on an ideal author and ideal reader joined through mutually accessible technical relations, putatively (albeit not practically) free from relying on privileged authorial testimony.^11^ Because the evidence for a mathematical claim is ideally contained in the exposition itself rather than produced in a laboratory or other setting inaccessible to a reader, conventions of mathematical authorship and attribution might be expected to emphasize credit over responsibility. Indeed, to the extent responsibility continued to matter in the history analyzed here, it is significant that charges of deception related to authorial identity were not connected to questions about the authenticity or validity of the mathematics beneath the byline. Authentication and integrity mattered outside of the usual social and philosophical matrix linking scientific evidence to scientific subjectivity – a disjunction evident in more recent efforts, such as the ORCID program of persistent digital identification, to authenticate scientific identities.^12^

With the story of Bourbaki and E.S. Pondiczery, a contemporary Bourbaki-inspired pseudonym, I shall advance two claims about authorship, fraud, and integrity in modern science. First, I claim that authorial integrity is a product, not a foundation, of the inevitably fictional co-construction of would-be authors and their would-be communities. This work of personification necessarily entails projection and always risks imposture. Scientific communities embrace or reject participants in turn by defining the bounds of acceptable representation. The primacy of these communal dynamics can be seen as well, in their absence, in a later attempt at pseudonymous projection by Kosambi, to whom this article returns in conclusion. Kosambi’s abortive personification of a mysterious figure, Sven Ducray, faltered for its lack of a consensual community to play along with the farce. For the two more successful pseudonyms of Bourbaki and Pondiczery, such collective representational labor constituted and authenticated legitimate – with Bourbaki, even monumental – authorial contributions to the mathematics discipline. Parodies defined communities of insiders who, by willingly or unwittingly playing along, made common cause with embodied and pseudonymous colleagues alike.^13^

Second, if authorial integrity is a fictive co-construction, so too is its converse of fraud. Faced with pseudonymous impostures, some mathematicians responded by defining their own communities of those *un*willing to play along by means just as fictional as those by which the pseudonyms’ supporters asserted their integrity. Authorial fictions, here, could not be exposed by reference to some regulatory truth, but only by constructing competing fictions. Fraud was a negotiated condition, dependent on local sociotechnical constructions of valid or invalid attempts to contribute to or profit from disciplinary formations.^14^ The boundaries between deception, parody, prank, and fraud were drawn and redrawn according to the social, institutional, and intellectual aims and values of different parties. The success or failure of each pseudonymous provocation hinged on dueling conceptions of the bounds of legitimate dissimulation, and the corresponding limits of who could be in on (or the butt of) a joke.

Following the layered representations and misrepresentations that animated Bourbaki and Pondiczery suggests that trust and verification may hinge less on faithful representation than on the socially constructed license to deceive fruitfully. Such deceptions are integral to modern scientific institutions, and can no more be rooted out than can authorship itself. Conventions of biography and bibliography, of validation and veneration, combined to support mathematicians’ participation in collective disciplinary formations that left ample space for play. Authorship was not an individual act but the outcome of communal relations of assertion and authentication – relations of a sort that continue to define legitimate participation in globally distributed scientific communities today. Here, integrity and fraud emerge as symmetric products of a common project of disciplinary fiction, defining both the individuals and communities that made up modern mathematics.

## Impersonation’s international infrastructures

That he did not generally speak for himself hardly made Bourbaki unique among famous names of mid-century mathematics. Secretaries routinely transcribed, composed, typed, and sent letters in mathematicians’ names. Correspondence was rife with excerpts, carbon copies, reported speech, and rumors. Most publications attributed a single author, but co-authorship was not unusual, and was understood to subsume unequal individual and shared efforts under a common attribution. However many authors adorned the byline, behind every paper lay a bevy of interlocutors and editors shaping words, formulations, and concepts.

The higher one’s status in the discipline, the more one’s name ultimately reflected such impersonations, which nucleated distributed collectives of scientific activity under a unifying fiction of personal agency.^15^ In this regard, establishing integrity in the sense of projecting the author as a coherent whole was always in tension with establishing integrity in the sense of transparently representing an author’s individual action. Projections of integral authorial identity were highly unstable, subject to refraction and revision according to changing social and intellectual goals and contexts.^16^ If Bourbaki’s integrity as a mathematician was an especially patent fiction, it depended on the consensual fictionality of every mathematician’s mediated identity.

Long segmented into relatively self-contained national and regional communities concentrated around a small number of dominant cities and institutions in Europe, mathematical research was by the early twentieth century only beginning to take a self-consciously international orientation, with periodicals leading the way.^17^ Promising mathematicians from outside the disciplinary metropoles might round out their training abroad, but (with some exceptions) even elite mathematicians had few needs or opportunities to travel extensively once established. Interwar philanthropy facilitated young mathematicians’ mobility to and between major centers, reinforcing those centers’ hegemonic position while extending their reach more decisively beyond national borders.^18^

With new opportunities to be interested in far-flung mathematicians came new challenges to keep up with their work, as well as new infrastructures to aid this pursuit.^19^ These allowed mathematicians to form and sustain scholarly communities with prohibitively distant colleagues, many of whom they would know primarily or exclusively by text. Large-scale projects of scientific bibliography dating to the latter half of the nineteenth century, including systematic international bibliographies in mathematics, raised the status and evaluative importance of articles and authorship.^20^ In 1931, the German publisher Springer launched an explicitly internationalist abstracting journal, the *Zentralblatt für Mathematik*, joined in 1940 by the American Mathematical Society’s *Mathematical Reviews* and in the 1950s by the Soviet Institute of Scientific and Technical Information’s *Referativny Zhurnal*.^21^

Together, these periodicals helped mathematicians across the globe to imagine something like an entire disciplinary literature to which presses and institutions around the world could contribute, unified despite the gaps and idiosyncrasies in individual library collections. Through reviews and citations, bylines multiplied across this expanded literature. As Cold War funding, jet travel, and other changes to mathematicians’ professional conditions let them personally travel much greater distances than their forebears,^22^ global communications infrastructures and the international institutions they supported and depended upon meant that mathematicians’ names traveled dramatically farther still. Bylines ran far ahead of bodies, and knowing people personally was, of necessity, supplemented and even displaced by knowing names. Each displacement demanded a cascading representation, a juncture for integrity or fraud that would test mathematicians’ emerging global institutions, norms, and practices.

## Cover stories

In the customary apparatus of academic publishing, cover letters bridge the personal, professional, and scholarly, allowing authors to assert identities, conflicts, and contexts that would not ordinarily appear in a formal academic text. Cover letters also, by this virtue, give cover to those who write them, offering a protected genre of quasi-confidential disclosure.

Under cover of cover letter, André Weil enlisted Élie Cartan, member of the Academy of Sciences and father of Bourbaki collaborator Henri Cartan, to communicate Bourbaki’s first mathematical publication to the Academy’s *Comptes Rendus* in 1935.^23^ According to Weil, Cartan was a witting accomplice, going so far as to consult with fellow academicians at a regular lunch group before endorsing the article Weil attributed to Bourbaki.^24^ The cover letter made a loophole out of a good-faith assumption, exposing the potential for absurdity in a system that relied on its constituents to play along. In Weil’s telling, he stressed to Cartan that the Academy member who communicated a paper was expected to vouch for its scientific merit but had no obligation to certify the authenticity of the paper’s author.

Unestablished scholars routinely drew on personal connections to figures like the elder Cartan to gain access to establishment journals like the *Comptes Rendus*. In principle, such gatekeepers protected the content of the mathematical literature – much as peer reviewers have been said to do in other scientific contexts – but in practice this system of personal referral was as much (or more) about endorsing authors as legitimate contributors to that literature. Communicating an article declared that its author ought to be heard – in the pages of the *Comptes Rendus* and implicitly beyond, including in bibliographic apparatuses such as the *Zentralblatt* or *Mathematical Reviews* that relied on each journal’s authorial authentication – not that its communicator agreed with every line. Weil invoked a specious ideal of scientific validation to license a subversive variation on its routine companion practice of personal validation.

Sending up the presumed familiarity shared within a scholarly community, Weil introduced Bourbaki in the cover letter with a knowing “Vous n’ignorez pas que. . .” – “You are not unaware,” or “As you know.” He situated the wayward professor at a café in the Paris suburb of Clichy, where he would fit stereotypically as a refugee from the vaguely Eastern-European invented country of Poldavia, recently “wiped off the map of Europe” by world events. Those very world events, combined with a longer history of Franco-Russian mathematical exchange, meant that a strange Slavic migrant was hardly an unlikely source of noteworthy results. Though no longer an active mathematician, Weil’s Bourbaki had previously lectured at the Royal University of Besse in Poldavia, a reference to Besse-en-Chandesse, France, site of the gathering where the Bourbaki collaborators conceived of the article. Bourbaki reportedly allowed Weil to browse his unpublished papers, whence the proffered communication.

The wider story of Poldavia, only partially evident in Weil’s letter, suggests further aspects of the cover letter genre’s work of disclosure, origin-making, and social justification. In his autobiography, Weil attributed Poldavia to another normalien prank, a decade before the parody that gave name to Bourbaki.^25^ This Poldavia began as a farcical government-in-exile whose members allegedly induced Paris sympathizers (in Weil’s recollection, with the same taunting intimacy of “You are no doubt familiar. . .”) to discuss the plight of the unfortunate nation at an open meeting, where they were ridiculed for their gullibility. That origin story may itself have been a retrospective cover story for Poldavia’s earliest historically verifiable appearance in the pun-dense 1929 missives by far-right-wing journalist Alain Mellet to dismiss soft-hearted rivals on the left.^26^ Mellet’s Poldavia had many imitators, including Belgian cartoonist Hergé, who dressed his hero Tintin in a false beard as Poldavian consul in a volume serialized just as Weil and collaborators were concocting their project under Bourbaki’s banner.

The cruel logic of Poldavian attribution could serve normaliens, the Action Française, or the Bourbaki conspirators just as well: in a political environment and academic discipline built on solidarity across imagined ties to unfamiliar people in strange-sounding places, the role of hardscrabble migrant could belong to anyone. To do anything other than take a pitiable biography at face value would be to invite charges of callousness, even inhumanity; to take it at face value, conversely, was to be exposed as naïve, credulous, provincial. The imposture’s credibility mattered little; its force derived less from the prospect of a successful duping^27^ than from the suggestion that the audience was susceptible to be duped. The only escape from this bind was to be in on the joke, to sympathize with the pranksters.

Here, in distilled form, was the politics of playing along. Through all manner of referrals and introductions, mathematicians formed ties to strangers on the basis of chains of trusting association, augmenting by compounded credulity. Parodying this genre served similar ends, propagating a joke to create a group of mathematicians that got it. Neither Weil, nor Kosambi before him, aimed with their transparently farcical biographies to admit Bourbaki into the mathematics profession; both aimed to assert their own places to friendly audiences by creating a joke they could share with people who mattered at the expense of people who did not.^28^ As Bourbaki became better known and the circle of those who knew not to take his biography seriously grew, biographical posturing became even more an inward-facing game than an outward-facing prank, a contest of artful allusions and clever inversions that affirmed membership in an in-group of consequence.

## Princeton Poldavians

When Weil visited Princeton in the first half of 1937, he inducted some of the younger mathematicians there into the Poldavian ruse, while also promoting Bourbaki’s mathematical ambitions. Among the newly minted Poldavians were Institute for Advanced Study postdoctoral scholar Frank Smithies, Princeton graduate-turned-postdoc Ralph Boas, and Princeton graduate student John Tukey.^29^ When Smithies and then Boas parted from Princeton, their and Tukey’s triangular correspondence formed a veritable trove of Poldaviana.^30^

Like their Bourbaki counterparts from France, the young Princeton mathematicians maintained an intense social life rife with in-jokes and wordplay. One favorite dinner-table punning game involved transmuting common mathematical turns-of-phrase into means of locating and caging a lion, for instance by inverting a spherical cage or traversing a space-filling curve.^31^ Inspired by Bourbaki’s entry in the *Comptes Rendus*, the Princeton conspirators decided to submit a compilation of their favorite methods pseudonymously to a leading American journal, the *American Mathematical Monthly*.^32^ Since the article was plainly farce, they attributed it to a plainly farcical author, H. Pétard – a reference to Shakespeare’s ‘hoisting’ line in *Hamlet*.

They explained the Pétard pseudonym in a cover letter signed with another pseudonym, E.S. Pondiczery.^33^ This latter pseudonym was a multi-layered tribute to Bourbaki, distorting the French-Indian city of Pondicherry with a Slavic spelling fit for a fellow Poldavian expatriate. Pondiczery’s initials came from the group’s casual, sardonic interest in extra-sensory perception, with the ‘S’ for Stanislaus and the ‘E’ later specified as Ersatz. To establish Pondiczery’s identity, the Princeton collaborators first contributed a half-page note in his name to the relatively unestablished-author-friendly collegiate “Questions, Discussions, and Notes” section of the *American Mathematical Monthly*, evidently without the editors’ awareness of Pondiczery’s pseudonymity.^34^

The business of fronting a pseudonym with another pseudonym required some ongoing misdirection for Pondiczery’s correspondence. Boas was Pondiczery’s principal ghostwriter for editorial matters.^35^ By the time the article appeared, however, Boas had decamped to Cambridge, England, to continue his postdoctoral studies. He left the job of “general forwarding agent” to his sister, Marie, who handled payments and reprints on Pondiczery’s behalf.^36^ Informing Tukey of the arrangement, Marie speculated that “if he [Pondiczery] continues to receive mail,” it might be better to route it to her brother by “send[ing] him [Pondiczery] back to Poldavia, address unknown, but stopping in Cambridge to see Ralph.” On matters of copyright for the lion-hunting article, the conspirators cut out the middleman and conducted correspondence as Pétard rather than Pondiczery.^37^

Social credit for Pondiczery and Pétard’s initial and ongoing work raised further considerations. Boas reported from Cambridge that “Pétard’s paper is attracting attention here,” generating “subdued chuckles . . . in the Philosophical Library.”^38^ When Tukey conveyed a word of praise for the article, Boas mused: “Since Pétard owes his existence to his friends, I don’t see how they can avoid accepting some of the responsibility for his works, alas.”^39^ In the two years following his debut, Pondiczery made several contributions to the “Gleanings Near and Far” column of mathematical miscellany in *The Mathematical Gazette*, while Pétard added to a Cambridge student society’s parodies.^40^ At the start of 1939, Boas proposed a new mathematical result to attribute to Pondiczery, but Smithies advised it was “too good for E.S.P.”^41^

The pen name also helped the conspirators avoid credit. One Tukey correspondent showed a Pétard reprint to a Rockefeller Foundation officer, who “didn’t see why they should publish such trash and [asked] did I know this man Pétard and, if so, was he as crazy as the stuff he wrote.”^42^ Boas replied that the officer’s “reactions indicate that the use of pen names was a very good thing. It is as well not to have even lunatics thinking you are crazy.”^43^ Paul Dirac, Boas gossiped, was not amused by Pétard’s “Dirac method.” The three gleefully reported on mistaken speculations about the article’s authorship.^44^

## Principled pseudonymity

Using pseudonymity to subvert some norms allowed the pseudonymers to sustain others. For Boas, one of the most important professional norms was engagement with the disciplinary literature as an author, referee, and reviewer. When the American Mathematical Society launched *Mathematical Reviews* in 1940, Boas contributed three reviews to each of its first two issues, and a total of twenty-eight that year, thirty in 1941, and thirty-four in 1942.^45^ In April 1942, fearing conscription after the U.S. entry into World War II, Boas found a job teaching mathematics to naval aviators in North Carolina.^46^ After discovering that his commanding officer did not want his staff publishing mathematics, Boas proposed to tell the *Mathematical Reviews* editor “that I have arranged for E.S.P. to take over my reviewing” and to give Tukey’s address so that the latter could forward materials to Boas.^47^

On the strength of Tukey’s recommendation, Boas mused that the editor “either suspects everything or nothing” and was “apparently going to play ball.”^48^ Over the first three issues of 1943, Boas’s reviews faded out and Pondiczery’s faded in, reversing again in the October issue. Pondiczery completed fourteen reviews that year to Boas’s twelve in 1943, and Boas roared back into form with forty entries under his own name in the 1944 volume.

The politics of playing along did not always require knowing who else was in the game. An editor who suspected everything or who suspected nothing would accommodate a pseudonym equally well; only one who suspected something but also had his doubts might raise trouble. So it was that Tukey found himself counseling the *Duke Mathematical Journal*’s J.M. Thomas, who confessed his puzzlement on learning that the S. Nooten whose manuscript Tukey had favorably reviewed was in fact the mathematician Warren Ambrose (himself an Institute for Advanced Study postdoc from 1939 to 1941 and Princeton instructor from 1941 to 1943).^49^ Though Thomas corresponded with Nooten at his given address in Princeton and was reassured by Tukey’s favorable referee report, Ambrose-qua-Nooten had raised in the editor “a suspicion that someone might be pulling our legs.”

Tukey pulled hard in the other direction. In reply to Thomas, he laid out three “principles concerning pseudonymous mathematical papers,” without revealing that he himself had first-hand experience with the genre.^50^ In cases like that of Bourbaki, “The use of a pseudonym by a continuing group personality is regarded as requiring no justification,” provided an editor at the journal knew “at least one member of the group” and could “sponsor the non-mathematical content of the papers.” For isolated papers, a group pseudonym “may or may not be advisable,” and was a matter for editorial discretion. Tukey did not give any examples, Pétard or otherwise, for this case. Finally, as with W.S. Gossett’s pseudonym Student behind the “Student’s *t*-test” in statistics, an individual might use a pseudonym with the approval of a fully witting senior editor who knows the actual author and circumstances. Indeed, Gossett’s 1907–9 circumstances were not so different from Boas’s wartime situation, both using pseudonyms to circumvent an employer’s prohibition on publication – although Gossett-qua-Student did so with the consent of his employer, the Guinness brewery. With both Bourbaki and Student, Tukey averred, “pseudonymous publication has, it seems to me, definitely advanced mathematics.” Ambrose-qua-Nooten’s paper, however, ultimately appeared under Ambrose’s name.

The very next month, editors at the same journal discussed what to do with a submission by one E.S. Pondiczery. Tukey had found his mark, and the Princeton Poldavians picked the Duke journal for Pondiczery’s first American research publication outside of the *American Mathematical Monthly* – after a short 1939 note in the journal of the Indian Mathematical Society and a four-year wartime lull – leading to his lone appearance in *Mathematical Reviews* as an author rather than reviewer. The journal’s Leonard Carlitz addressed a letter to Pondiczery, and though the letter itself does not appear to have been preserved, Tukey’s reaction indicates that it raised the matter of pseudonymity explicitly. Accordingly, Tukey sent Carlitz two replies, one in Pondiczery’s name – advising Carlitz that Pondiczery would like the article to appear under Pondiczery’s own name and that he had shared the letter with Tukey, who might write separately – and one under Tukey’s own name.^51^

Tukey’s autograph letter referred Carlitz to his advice to Thomas about Nooten. In accordance with that advice, and without revealing his part in the Pondiczery scheme, Tukey wrote:

I am prepared to say that Pondiczery is a continuing group personality, that I have seen some of his articles in other journals, and that I know some members of the group well enough to be very willing to stand sponsor for the non-mathematical content of any papers he may offer for publication.

Adding “I doubt that he will become another Bourbaki,” Tukey suggested Pondiczery was “much more likely to benefit mathematics than to hinder it.” Unlike Nooten’s, Pondiczery’s paper appeared later that year under the pseudonym.^52^

While pseudonyms clearly gave editors pause when they knew enough to spot them, the prevailing question was not *whether* they could be a boon to mathematics, but under what circumstances. Names, in a publishing profession, indexed credit, reputation, and responsibility alongside social, professional, and intellectual ties. Permissible misrepresentations were governed by higher principles of participation, collegiality, solidarity, and insight, to be adjudicated with confidential assurances shared among credentialed elites. Deceit could be virtuous if committed honestly.

## Cited, unseen

To an unwitting reader – that is, at first, to all but the conspirators themselves and the small circle with whom they shared their joke – Pondiczery and Bourbaki blended seamlessly into the flood of strange names that inundated mathematical libraries wherever professional mathematicians could be found. As early as 1941, Boas noted to Tukey the mass of obscure Soviet journals that, thanks to the new *Mathematical Reviews*, were now not just available but imposed on his disciplinary field of view.^53^ Boas took over the editorship of *Mathematical Reviews* at the start of a postwar flood, not just from the Soviet Union: after a norm of around 2,000 *total* papers annually, he gasped in February 1946 at a delivery of 400 new papers from Japan alone.^54^ That October, he averred to Smithies that “material is accumulating at a frightening rate.”^55^

To keep up, editors and researchers redoubled their reliance on a framework of attribution and circulation driven by authors’ bylines, supported in libraries and editorial offices by the powerful indexical technology of the card catalog. At *Mathematical Reviews*, cards recorded both the mathematical literature and the addresses and competencies of prospective reviewers. During Boas’s tenure as editor, he used the latter reviewer file to assign a paper in Gaelic to the lone reviewer who declared competence in the language, whereupon he learned that the paper’s byline was a Gaelicized name from the presumptive reviewer.^56^ While not strictly a pseudonym, the author’s Gaelic name may as well have been one, and the same bureaucratic and attributory practices that accommodated such variants also accommodated proper pseudonyms like Bourbaki and Pondiczery. Editors habituated themselves to dealing with unfamiliar addresses, trusting in the institutional machinery of international mathematics to hold everything together. Where editors were in the know, such apparatus need not extend to colleagues’ fabrications: Bourbaki’s fictionalized addresses, like the portmanteau Nancago of Nancy and Chicago, did not filter into *Mathematical Reviews*, nor did Bourbaki ever join Pondiczery as a reviewer.^57^

Under Boas’s postwar editorship, Bourbaki’s textbooks drew prominent and sympathetic treatment in *Mathematical Reviews*, and Boas continued to promote Bourbaki in reviews after his tenure.^58^ As a reviewer himself, however, he did not treat Bourbaki as an ordinary author, twice in 1949 outing the name as a pseudonym. The first was in an annual review of mathematics for the *Encyclopaedia Britannica*, over which Bourbaki lodged a complaint with the encyclopedia’s editors, who did not know to treat it as a joke and asked Boas to support his attribution. The second was in a *Mathematical Reviews* entry quoting André Delachet’s description of Bourbaki as “the polycephalic mathematician” – a quotation Boas found in the course of defending his *Britannica* entry from Bourbaki’s protests and presumably determined ought to be shared more widely.^59^

In the first decade of *Mathematical Reviews*, Bourbaki’s name appeared a total of twenty-nine times: eleven as an author and the rest in citations in other reviews. Reviews for five of the first six volumes of Bourbaki’s textbook fell to Samuel Eilenberg, who went on to join the Bourbaki collaboration in 1950. For the most part, Eilenberg treated Bourbaki as an ordinary name in the mathematical literature, marking it as a pseudonym only in his first review. As citation, Bourbaki filtered into *Mathematical Reviews* when reviewers noted prominent citations from the articles under consideration – articles themselves typically written by Bourbaki collaborators who featured Bourbaki as a means of sideways self-promotion. In six reviews, Bourbaki collaborator Jean Dieudonné insisted that the pseudonym’s primacy or priority had been neglected in the reviewed work.

Where Pondiczery was most often a pseudonym of convenience, with his biography an ongoing social game, the comparatively systematic Bourbaki project placed greater organizational importance on the pseudonym’s byline while drawing attention away from biographical details that were incidental to the project’s ongoing operation. In practice, Bourbaki’s impersonators treated their collective as an open secret, breaking in and out of character where needed, for instance, in a grant application or career move. Behind the scenes, as opposed to in the mathematical literature, Bourbaki’s personhood was often fleeting. Though more famous for his outlandish biography, his bibliography mattered most.

## From fiction to fraud

Playing along with Bourbaki or Pondiczery in the mathematical literature entailed different demands and called upon different norms and practices from playing along with the pseudonyms as biographical figures. These latter exigencies, invoked outside the matrix of principles that legitimated routine pseudonymity in publication, let the pseudonyms nucleate challenges to the institutional orders of international mathematics. One glimpses this transgressive potential in Boas and Tukey’s 1942 speculation about getting Pondiczery into the reference volume *American Men of Science*.^60^ The line between such a venture and article-oriented indices like *Mathematical Reviews* was thin but significant: both were guides to who and what mattered in the discipline, but the respective emphases on the men or the papers made the difference between earnest participation in a collective publishing enterprise and gnomic parody of indexers’ indifference to their subjects’ humanity or reality.

First in 1948 and again in 1949, Bourbaki applied for membership in the American Mathematical Society (AMS), and was rebuffed both times.^61^ The 1948 application arrived from the University of Chicago not long after Weil joined its mathematics faculty, claiming Bourbaki as a ‘nominee’ covered by the department’s institutional membership and bearing a signature closely resembling two specimens attributable to Weil from a decade prior.^62^ The rest of the application, calling for various biographical attestations, mixed the vaguely plausible with the plainly parodic. Bourbaki was currently employed as a “Fellow of the Rockefeller Foundation” and could be reached at the Institut de Mathématique at the University of Nancy, a lightly fictionalized address that adopted Bourbaki’s textbooks’ characteristic singular variation on mathematic(s). From 1910 to 1919, he was “Professor, Royal Poldavian University,” and was born in Cucutemi, Poldavia, a play on the real village of Cucuteni, Moldavia, on the non-leap year date of 29 February 1885. His textbooks appeared by indirect reference to his employment as “Scientific Advisor, Hermann Publishing Co., 1934-.” AMS Secretary John Kline did not allow the application to be taken seriously.^63^

Bourbaki’s second application, dated 15 December 1949, would be harder to dismiss.^64^ Though no historian has unearthed the paperwork,^65^ there is no reason to doubt that Bourbaki had entered the rolls of the Société Mathématique de France (SMF) earlier that year, and so was eligible under a 1946 reciprocity agreement with the AMS to join the American society as well.^66^ The new application was entirely handwritten, and the handwriting appears to support Beaulieu’s attribution to Jean Dieudonné, of Nancy.^67^ Dieudonné’s Bourbaki was born in Cucuteni, Moldavia, on 12 December 1886, elected to the Royal Academy of Poldavia in 1917 and the SMF in 1949, and presently employed at Dieudonné’s institute in Nancy. His 1910 doctorate came from Kharkov University, a real institution but sufficiently remote as to be unverifiable. Previous employers included the real Dorpat University, in Estonia, and the fictional Zorngahr College, an apparent portmanteau of German algebraist Max Zorn and Weil’s first employer in Aligarh.

Kline took offense at once again being the butt of a Bourbaki prank, considering it an affront to the AMS’s dignity.^68^ His response is telling for its procedural assumptions. That Bourbaki was a pseudonym was well-known at this point to a great many AMS members (albeit not the two who, as a matter of bureaucratic routine, cosigned his application^69^), but Kline did not simply do as he had done the first time and rule Bourbaki ineligible on those grounds. Rather, he expended a special effort to demonstrate Bourbaki’s application as not just fictional but fraudulent.

This subtle distinction reflected the bureaucratic logic of the far-reaching international ties Kline had, by then, been building for nearly a decade as AMS Secretary. Because he had to take unverifiable biographies at face value in the daily operation of a society that now embraced personally unknown mathematicians from far away, any principle used to reject Bourbaki must also derive from taking his application at face value. The primacy of face-value representations in international mathematical institutions and infrastructures had, by this point, become axiomatic. Indeed, as one of the mathematicians who initially received and cosigned the application observed, to deviate at all from the “essentially clerical” role of processing the applications as submitted, and especially to deny a member ostensibly in good standing of the SMF the benefits of the reciprocity agreement, might invite “international complications.”^70^

Because Dieudonné could not consult Weil’s prior application and relied instead on their shared but fragmentary stock of Bourbaki lore for the second application’s particulars, significant discrepancies could hardly be avoided. Kline promptly had a secretary compose a two-column table comparing the biographical details.^71^ The very fluidity of Bourbaki’s biography, in other contexts, was part of the joke: few biographies of legendary figures are fixed with rigid certainty, and inconsistent rumors, if anything, added verisimilitude to a past shrouded in mystery. This conceit only worked, however, for biographies framed as second-hand reports, precisely the biographical genres that dominated the discourse of communities of insiders built around a shared joke. On an application form, Kline could take the biographical details as personal attestations coming from Bourbaki himself, and so deduce – while still taking everything at face value – that the attesting person named on both forms must have lied on at least one of them.

Most of the biographical claims were, in practice, unverifiable. Kline could neither write to far-off universities or possibly defunct academies, nor assert with incontestable authority that they did not exist – notwithstanding his justified certainty that the claims were invented. Bourbaki’s Rockefeller Fellowship, however, could be easily assayed from within Kline’s own professional network. He wrote to the foundation’s Warren Weaver on the pretense of checking Bourbaki’s fellowship history, but really as a chance to vent his frustration over the undignified behavior of the French upstarts. Even Bourbaki’s discrepant signatures earned a remark, having deteriorated from that “of a determined man” in the first application to a “cramped” and “infantile” one in the second.^72^ Weaver endorsed Kline’s evaluation of the prank as “quite childish” and noted that his foundation had supported Bourbaki but not, of course, as an individual fellow. Aware of Weil’s role in the collaboration but not of his involvement in the application, Weaver supposed that as a “responsible member of the group” Weil could broker a more reasonable approach.^73^ As it did for Tukey in his exchange with the *Duke Mathematical Journal*, the selective anonymity of Weil’s pseudonym-building let him maintain a reputation as a responsible broker while avoiding responsibility for potentially less-reputable impostures.

Kline’s strategy of undermining the application at face value proved effective for most members of the AMS Executive Council whom he consulted.^74^ Only Einar Hille, recent AMS president and a supporter of the Bourbaki enterprise, found Kline’s approach unconvincing.^75^ Hille advised that “a formalistic attitude” played right into the Bourbaki provocation, “and standing on our dignity will not get us anywhere and may expose us to ridicule.” Favoring the kinds of disembodied contributions to mathematics reflected in journals, reviews, and lore, Hille asserted that, ultimately, “a good fictitious character lives more intensely and a good deal longer,” and he had no doubt “that N. Bourbaki has made a stronger imprint on present day mathematics and his fame will last longer than that of most of present members of our Society and it behooves us of taking cognizance of this fact.” Hille thought the AMS should welcome the chance to join in the joke, but Kline stuck to his formalism and recommended the AMS Council reject the application.^76^

The Council carried out the recommendation in December 1950, writing that “it is well known that Bourbaki is not an individual but rather a group of distinguished mathematicians” and offering the group an institutional membership.^77^ The AMS had to pretend to take the application as a sincere attempt to gain membership from an individual in order to challenge the premise that Bourbaki should be eligible. That is, they rejected the application because they knew it to be a farce, but their procedure for this rejection depended on an elaborate performance of believing the application to be genuine. Demonstrating fraud required fictions just as disingenuous and implausible as the alleged fraud itself.

Their reliance on formal criteria provoked a straightforward response from Dieudonné on Bourbaki’s behalf, reminding the AMS that “it is the clear meaning of the first paragraph of the reciprocity agreement . . . that the A.M.S. had no right to scrutinize” the application.^78^ Where the AMS tried to deflate the prank by taking it seriously, Dieudonné insisted that the Americans must have been joking, and that Bourbaki had taken their (otherwise implicitly offensive) rejoinder in that spirit. To the offer of institutional membership, Dieudonné countered by suggesting institutional membership for the University of Nancago. If AMS officers thought they could win by playing along, Dieudonné would change the game.

## Conclusion: Playing along

It was only a matter of time before Kosambi, who brought Bourbaki-the-mathematician into the world, birthed his own pseudonym. Like most of the Bourbaki story, this part runs backward: Nicolas Bourbaki’s history was an elaborate and consequential farce, repeated in Kosambi’s pseudonym Sven Ducray as tragedy. The name recalls the mathematician’s affectionate nickname for his family’s overfed dog: *Dukker* (Marathi for pig), with the first name from *Svana* (Sanskrit for dog).^79^ Ducray began writing and publishing at the tail end of Kosambi’s once-bright mathematical career, as Kosambi tried and failed to convince friends and colleagues at home and abroad that he had proven the Riemann Hypothesis, to this day one of the most important open problems in mathematics and a repeated subject of claimed proofs from established and fringe mathematicians alike. In 1958, as Kosambi began circulating his proofs, Weil pled with him to keep them to himself and questioned his mathematical acumen.^80^

A narrow slip of paper tucked among a collection of Kosambi and Ducray offprints in Kosambi’s archive, now preserved in New Delhi, gives the pseudonym’s only biography of any detail:

Little is known about Sven Ducray except that he emerged as a number-theorist during long convalescence in India after a voyage over the backlands of China and Burma. The constantly changing, unpredictable date-line of his rare letters, from places right off the map, indicate neither fixed address nor connection with any academic institution. Though our author travelled around the world on some west European passport, he neither claims nor disclaims kinship with the Ducray whose name appears briefly in the annals of the French Revolution.^81^

In the mathematical literature, Ducray teased with circumlocutions, such as the acknowledgment that “This paper would not have been possible without the constant labour of Prof. D. D. Kosambi” and, elsewhere, that “The full extent of my debt to Prof. D. D. Kosambi will some day be revealed, as well as the reason for not making better acknowledgement here.”^82^

Ducray’s papers detailed arguments that Kosambi advanced and developed in the wake of 1958 through an extensive correspondence with leading mathematicians in Europe and North America, whose initial incredulity wore into exasperation or exhaustion. Helped along by Kosambi’s promotion, the papers received ordinary notice in both *Mathematical Reviews* and the *Zentralblatt für Mathematik*, with reviews signaling apparent errors.^83^ Representing himself as Ducray’s mentor, Kosambi corresponded with at least one publisher on Ducray’s behalf and traded on his own prestige to see Ducray’s paper to print.^84^ In addition to publishing as Ducray, Kosambi circulated unpublished drafts of Ducray’s work to other mathematicians, including some with whom Kosambi had longstanding friendly correspondence of his own, without ostensibly revealing Ducray’s pseudonymity.^85^ In epistolary global mathematics, only Kosambi knew Ducray was a dog.^86^

Ostracized in his national mathematical community and courting discredit abroad, Kosambi concocted Ducray in his refusal to play along. Without a community of co-conspirators identified with Kosambi’s quixotic assault on the Riemann Hypothesis, Ducray floated through letters, papers, and reviews as an unremarkable name unable to inspire the winking solidarity that marked the likes of Bourbaki for greatness but equally indistinguishable from the masses of faceless entrants to a burgeoning global profession. The pseudonyms of modern mathematics have been most powerful at their least deceptive. For the ever-widening circle who knew that Bourbaki was a pseudonym, playing along was a deliberate decision about one’s mathematical sociability. Younger mathematicians who thrilled to Bourbaki’s postwar textbooks alike embraced Bourbaki-the-byline as one more way to align themselves with the mathematical vanguard. Making up people made up communities, movements, and generations.

Mid-century mathematicians’ impersonations followed the channels and infrastructures by which non-pseudonymous entrants to the profession established themselves: in short articles advanced by allies, in letters and reviews, in professional associations. Impersonation was a serious matter, defining the conditions of participation in a discipline where people depended on long-distance personal connections made and maintained multifariously. Defending those conditions from farce and imposture was also a serious matter, requiring fictions of its own. Neither legitimate nor fraudulent contributions to mathematics can be understood solely (or even primarily) in terms of the actions of individual perpetrators. Authenticity and fraud were both the products of collective fiction within mathematicians’ social institutions, reliant on their changing infrastructures, available alike to man and myth.

## Notes

1. Liliane Beaulieu, *Bourbaki: Une histoire du groupe de mathématiciens français et de ses travaux (1934–1944)* (Ph.D. Dissertation, Université de Montréal, 1989), pp.279–81; André Weil, *The Apprenticeship of a Mathematician*, trans. by Jennifer Gage (Basel: Birkhäuser, 1992 [1991]), p.101.

2. D.D. Kosambi, “On a Generalization of the Second Theorem of Bourbaki,” *Bulletin of the Academy of Sciences, Uttar Pradesh* 1 (1931): 145–147, in Ramakrishna Ramaswamy (ed.), *D.D. Kosambi: Selected Works in Mathematics and Statistics* (New Delhi: Springer, 2016), pp.55–7.

3. The real Russian work was L. Lusternik and L. Schnirelmann, *Topological Methods in the Calculus of Variations* (Moscow: Research Institute of Mathematics and Mechanics, 1930), and Kosambi’s interest in its translation was shared by, e.g., R.C. Archibald, “New Mathematical Periodicals,” *The American Mathematical Monthly* 39 (1932): 185–7, 186. Reviewing Kosambi’s paper in the *Jahrbuch über die Fortschritte der Mathematik* (JFM 58.0764.01, accessed via the zbMATH database), Dutch mathematician J.A. Schouten mistook Weil for German mathematician Hermann Weyl and regretted “that it is not explicitly said what comes from Bourbaki and of what the generalization consists, since the mentioned Russian work is not accessible to most colleagues.” The alphabet joke claimed Bourbaki was shot alongside “other members of the ‘Russko-Angliskii Slovar’ [‘Russian-English Dictionary’]” after an “affair” named for a distinctive character, the soft sign Ь.

4. Liliane Beaulieu, “A Parisian Café and Ten Proto-Bourbaki Meetings (1934–1935),” *The Mathematical Intelligencer* 15 (1993): 27–35; Leo Corry, “Writing the Ultimate Mathematical Textbook: Nicolas Bourbaki’s *Élements de mathématique*,” in Eleanor Robson and Jacqueline Stedall (eds.), *The Oxford Handbook of the History of Mathematics* (Oxford: Oxford University Press, 2009) pp.565–88.

5. Liliane Beaulieu, “Bourbaki’s Art of Memory,” *Osiris* 14 (1999): 219–51. R.P. Boas, “Bourbaki, Nicolas,” in Charles Gillespie (ed.), *Dictionary of Scientific Biography* (New York: Scribner, 1970) vol. 2, pp.351–3; R.P. Boas, “Bourbaki and Me,” *The Mathematical Intelligencer* 8 (1986): 84. See also Leo Corry, “Bourbaki, Nicolas,” in Noretta Koertge (ed.), *New Dictionary of Scientific Biography* (Detroit: Scribner, 2008) vol. 19, pp.363–6.

6. Cf. Buhm Soon Park, “Making Matters of Fraud: Sociomaterial Technology in the Case of Hwang and Schatten,” *History of Science* 58 (2020): 393–416.

7. E.g. Steven Shapin, *A Social History of Truth: Civility and Science in Seventeenth-Century England* (Chicago: University of Chicago Press, 1994).

8. Mario Biagioli, “The Instability of Authorship: Credit and Responsibility in Contemporary Biomedicine,” *The FASEB Journal* 12 (1998): 3–16.

9. Ibid., pp.6–9; Mario Biagioli, “Rights or Rewards? Changing Frameworks of Scientific Authorship,” in Mario Biagioli and Peter Galison (eds.), *Scientific Authorship: Credit and Intellectual Property in Science* (New York: Routledge, 2003), pp.253–79.

10. Hugh Gusterson, “The Death of the Authors of Death: Prestige and Creativity among Nuclear Weapons Scientists,” in Biagioli and Galison, *Scientific Authorship*, pp.281–307 (note 9).

11. Andrew Warwick, “A Very Hard Nut to Crack: or Making Sense of Maxwell’s *Treatise on Electricity and Magnetism* in Mid-Victorian Cambridge,” in Biagioli and Galison, *Scientific Authorship*, pp.133–61 (note 9); Michael J. Barany, *Proof and Its Putting: Mathematics, Rigor, and Testimony* (AB Thesis, Cornell University, 2008), http://mbarany.com/CornellThesis.pdf; Brian Rotman, “Toward a Semiotics of Mathematics,” *Semiotica* 72-1/2 (1988): 1–35.

12. One reviewer for this article lamented the threat ORCID posed to projects like those discussed here, but as of this writing even Nicolas Bourbaki has an ORCID identity, albeit without an actively maintained profile, https://orcid.org/0000-0003-0056-1249 (accessed 2020).

13. On the constitutive importance of knowingly playing along, cf. Kara Swanson, “Biotech in Court: A Legal Lesson on the Unity of Science,” *Social Studies of Science* 37 (2007): 357–84.

14. Cf. Park, “Making Matters of Fraud” (note 6).

15. Cf. Hélène Mialet, *Hawking Incorporated: Stephen Hawking and the Anthropology of the Knowing Subject* (Chicago: University of Chicago Press, 2012); Peter Galison, “The Collective Author,” in Biagioli and Galison, *Scientific Authorship*, pp.325–55 (note 9).

16. Frédéric Brechenmacher, “L’auteur en mathématique: l’exemple d’Évariste Galois (1829 S),” *L’Archicube* 19 (2015): 118–25. Brechenmacher notes the Bourbaki collaborators’ role in retrospectively revising Galois’s authorial identity as part of their own mathematical program. Cf. Anne-Sandrine Paumier and David Aubin, “Polycephalic Euclid? Collective Practices in Bourbaki’s History of Mathematics,” in Volker R. Remmert, Martina R. Schneider, and Henrik K. Sørensen (eds.) *Historiography of Mathematics in the 19th and 20th Centuries* (Cham: Birkhäuser, 2016), pp.185–218.

17. See Karen H. Parshall and Adrian C. Rice (eds.) *Mathematics Unbound: The Evolution of an International Mathematical Research Community, 1800–1945* (Providence: American Mathematical Society, 2002); Michael J. Barany, *Distributions in Postwar Mathematics* (Ph.D. Dissertation, Princeton University, 2016), pp.5–14, 25–86.

18. E.g. Reinhard Siegmund-Schultze, *Rockefeller and the Internationalization of Mathematics Between the Two World Wars: Documents and Studies for the Social History of Mathematics in the 20th Century* (Basel: Birkhäuser, 2001). Several Bourbaki collaborators were early recipients of Rockefeller-funded fellowships.

19. See Michael J. Barany, “Abstract Relations: Bibliography and the Infra-structures of Modern Mathematics,” *Synthese*, advance online publication (2020), DOI: 10.1007/s11229-020-02683-3.

20. Alex Csiszar, “How Lives became Lists and Scientific Papers became Data: Cataloguing Authorship during the Nineteenth Century,” *British Journal for the History of Science* 50 (2017): 23–60.

21. See Reinhard Siegmund-Schultze, “‘Scientific Control’ in Mathematical Reviewing and German-U.S.-American Relations between the Two World Wars,” *Historia Mathematica* 21 (1994): 306–29.

22. See Michael J. Barany, “Fellow Travelers and Traveling Fellows: The Intercontinental Shaping of Modern Mathematics in Mid-Twentieth Century Latin America,” *Historical Studies in the Natural Sciences* 46 (2016): 669–709.

23. Beaulieu, “Memory,” 238–9 (note 5); Beaulieu, *Une histoire*, 284–6 (note 1). Nicolas Bourbaki, “Sur un théorème de Carathéodory et la mesure dans les espaces topologiques,” *Comptes Rendus des Séances Hebdomadaires de l’Académie des Sciences* 201 (1935): 1309–11.

24. Weil, *Apprenticeship*, pp.101–2 (note 1).

25. Weil, *Apprenticeship*, p.102 (note 1); Michèle Audin, “La Vérité sur la Poldévie,” July 2009. http://oulipo.net/fr/la-verite-sur-la-poldevie (2017).

26. The French Wikipedia page for *Poldévie* is a well-documented guide to Mellet’s version of the hoax. https://fr.wikipedia.org/w/index.php?title=Pold%C3%A9vie&oldid=156652915 (2019).

27. But see Beaulieu, *Une histoire*, p.286, n. 177 (note 1), for a prominent mathematician’s testimony to having been duped by the 1935 byline.

28. On the contributions of such obviously farcical fictions to the consolidation and operation of actual communities and collaborations, see Beaulieu, “Memory” (note 5) and Beaulieu, “Jeux d’esprit et jeux de mémoire chez N. Bourbaki,” in Pnina G. Abir-Am (ed.), *La mise en mémoire de la science: pour une ethnographie historique des rites commémoratifs* (Amsterdam: Editions des archives contemporaines, 1998), pp.75–123.

29. See Beaulieu, *Une histoire*, p.286 (note 1); Ralph P. Boas, Jr., “Autobiographical Essay,” in Gerald L. Alexanderson and Dale H. Mugler (eds.), *Lion Hunting & Other Mathematical Pursuits: A Collection of Mathematics, Verse and Stories by Ralph P. Boas, Jr.* (Washington, DC: Mathematical Association of America, 1995), pp.1–24, 9–10. Boas omits Tukey, while Beaulieu implies that Bourbaki collaborator Claude Chevalley was a part of the group. The latter arrived at the Institute for Advanced Study over a year after Weil’s departure, in time to have enjoyed the fallout from the group’s pranks.

30. John W. Tukey Papers, American Philosophical Society, Philadelphia, PA (hereafter “Tukey Papers”), “Boas, Ralph Philip” (hereafter “Boas–Tukey Correspondence,” Boas to Tukey unless otherwise specified); Tukey Papers, “Smithies, Frank” (hereafter “Smithies–Tukey Correspondence,” Smithies to Tukey unless otherwise specified); Papers of Frank Smithies, St. John’s College Library, University of Cambridge (hereafter “Smithies Papers”), quotations by permission of the Master and Fellows of St. John’s College, Folder A10 (hereafter “Boas–Smithies Correspondence,” Boas to Smithies unless otherwise specified); Smithies Papers, Folder A66 (hereafter “Tukey–Smithies Correspondence,” Tukey to Smithies unless otherwise specified).

31. See Michael J. Barany, “Integration by Parts: Wordplay, Abuses of Language, and Modern Mathematical Theory on the Move,” *Historical Studies in the Natural Sciences* 48 (2018): 259–99, 276–82.

32. H. Pétard “A Contribution to the Mathematical Theory of Big Game Hunting,” *American Mathematical Monthly* 45 (1938): 446–7.

33. Boas, “Autobiographical,” p.10 (note 29).

34. E.S. Pondiczery, “A Function-Theoretical Paradox,” *American Mathematical Monthly* 45 (1938): 307.

35. E.g. Moulton to Boas, 5 October 1938, Boas–Tukey Correspondence; see also 19 July 1938, Boas–Smithies Correspondence.

36. M. Boas to Tukey, 12 October 1938, Tukey Papers, “Boas, Marie” folder. On reprints and payments, see also M. Boas to Tukey, 7 December 1938, and Tukey to M. Boas, 11 December 1938, “Boas, Marie” folder; R.P. Boas to Tukey, 19 September 1938, M. Boas to Tukey, 18 December 1938, and Tukey to M. Boas, 22 January 1939, Boas–Tukey Correspondence. Historians of science may recognize Marie as Marie Boas Hall, distinguished historian of the Scientific Revolution.

37. E.g. 1 June 1939, Boas–Tukey Correspondence.

38. 5 November 1938, Boas–Tukey Correspondence. See also 30 January 1940, Boas–Smithies Correspondence.

39. 10 November1938, Boas–Tukey Correspondence.

40. See 28 February 1940, Boas–Smithies Correspondence.

41. Boas to Smithies, 1 January 1939, Smithies to Boas, 9 January 1939, Boas–Smithies Correspondence.

42. 4 December 1938, Tukey–Smithies Correspondence.

43. 17 December 1938, Boas–Tukey Correspondence. On this Rockefeller officer’s own authorial practice and its relation to the funding and institutional order of mid-century mathematics, see Michael J. Barany, “The Officer’s Three Names: The Formal, Familiar, and Bureaucratic in the Transnational History of Scientific Fellowships,” in John Krige (ed.) *How Knowledge Moves: Writing the Transnational History of Science and Technology* (Chicago: University of Chicago Press, 2019), pp.254–80.

44. E.g. 3 December 1939, Boas–Tukey Correspondence; 12 December 1939, 4 September 1944, Boas–Smithies Correspondence.

45. Figures from the American Mathematical Society’s MathSciNet database.

46. 26 April 1942, Boas–Tukey Correspondence. Boas, “Autobiographical,” p.14 (note 29).

47. Undated circa 1942, Boas–Tukey Correspondence.

48. Boas to Tukey, 28 September 1942; see also Tukey to Boas, 2 October 1942, Boas–Tukey Correspondence. Cf. Boas to Tukey, 17 January 1943, where Boas reports he will “continue to let ESP do my reviewing.”

49. Thomas to Tukey, 27 July 1944, Tukey Papers, “Duke Math Journal” folder (hereafter “Tukey-DMJ”).

50. Tukey to Thomas, 11 August 1944, Tukey-DMJ. After the present article’s acceptance, a new article became available online that offers a striking current parallel to Tukey’s missive on acceptable pseudonimity, even mentioning Tukey’s examples of Student and Bourbaki: Bart Penders and David M. Shaw, “Civil disobedience in scientific authorship: Resistance and insubordination in science,” *Accountability in Research* 27 (2020): 347–371; see esp. pp.7–8. Cf. Max Liboiron et al., “Equity in Author Order: A Feminist Laboratory’s Approach,” *Catalyst* 3 (2017), DOI: 10.28968/cftt.v3i2.28850.

51. Tukey to Carlitz, 25 September 1944, Tukey-DMJ; Tukey to R. and M. Boas, 25 September 1944, Boas–Tukey Correspondence. See also Carlitz to Tukey, 2 October 1944, Tukey-DMJ.

52. E.S. Pondiczery, “Power Problems in Abstract Spaces,” *Duke Mathematical Journal* 11 (1944): 835–7.

53. Postcard postmarked 2 March 1941, Boas–Tukey Correspondence.

54. 12 February 1946, Boas–Tukey Correspondence.

55. 14 October 1946, Boas–Smithies Correspondence.

56. Boas, “Autobiographical,” p.16 (note 29). 11 June 1949, Boas–Smithies Correspondence.

57. E.g. N. Bourbaki, “Foundations of Mathematics for the Working Mathematician,” *Journal of Symbolic Logic* 14 (1949): 1–8; idem, “The Architecture of Mathematics,” trans. Arnold Dresden, *The American Mathematical Monthly* 57 (1950): 221–32. See Liliane Beaulieu, “Quand Nancy s’appelait Nancago,” *L’Est Républicain*, 15 June 2011. https://www.estrepublicain.fr/education/2011/06/15/quand-nancy-s-appelait-nancago.

58. E.g. Stone to Boas, 27 September 1951, with Boas’s manuscript reply, Folder 15, Box 38. Papers of Marshall Stone, John Hay Library, Brown University, regarding a systematic Bourbaki review for the *Bulletin of the American Mathematical Society*.

59. Boas, “Bourbaki and Me” (note 5); Paul R. Halmos, “Nicolas Bourbaki,” *Scientific American* 196 (1957): 88–99, 93–94; 11 December 1949, Boas–Smithies Correspondence.

60. 20 December 1941 and 1 March 1942, Boas–Tukey Correspondence.

61. Everett Pitcher, *A History of the Second Fifty Years: American Mathematical Society, 1939–1988* (Providence: American Mathematical Society, 1988), pp.159–62; Beaulieu, “Nancago” (note 57).

62. Records of the American Mathematical Society, John Hay Library, Brown University, Box 34, Folder 57 (AMS records hereafter noted as “AMS” followed by the box and folder number). Weil’s Bourbaki signatures are in Smithies Papers, Folder A69, and reproduced (from a different source) in Maurice Mashaal, *Bourbaki: A Secret Society of Mathematicians*, trans. Anna Pierrehumbert (Providence: American Mathematical Society, 2006 [2002]), p.28.

63. Kline to Hildebrandt, 16 January 1950, AMS 36-11.

64. AMS 36-11, Application for Membership, 15 December 1949, and Thrall to Hull, 5 January 1950.

65. But see Michèle Audin (ed.) *Correspondance entre Henri Cartan et André Weil (1928–1991)* (Paris: Société Mathématique de France, 2011), p.600, n. 296.2.

66. AMS 37-33, “Reciprocity Agreement between the American Mathematical Society and the French Mathematical Society.”

67. Beaulieu, “Nancago” (note 57).

68. AMS 36-11, Kline to Hildebrandt, 16 January 1950; Kline to Thrall, 6 February 1950.

69. AMS 36-11, Hildebrandt to Kline, 20 January 1950, Thrall to Kline, 27 February 1950.

70. AMS 36-11, Thrall to Kline, 27 February 1950.

71. AMS 36-11, “Information given about Nicolas Bourbaki.”

72. AMS 36-11, Kline to Weaver, 6 March 1950.

73. AMS 36-11, Weaver to Kline, 7 March 1950.

74. AMS 36-11, Kline to Hille, Walsh, and Martin, 6 March 1950; Walsh to Kline, 8 March 1950; Martin to Kline, 9 March 1950.

75. AMS 36-11, Hille to Kline, 8 March 1950.

76. AMS 36-11, Kline to Hille, MacLane, Martin, and Walsh, 11 April 1950.

77. AMS 37-15, Begle to Foures, 27 February 1951.

78. AMS 37-15, Dieudonné to Begle, 20 July 1951.

79. Ramaswamy, *D.D. Kosambi*, p.12 (note 2). Ramaswamy identifies three earlier Kosambi aliases, none with the biographical trappings of the later pseudonym. Cf. the multiplication of pseudonyms and authorial tactics attributed to disciplinarily marginalized English historian A.D. Harvey in Eric Naiman, “When Dickens met Dostoevsky,” *The Times Literary Supplement*, 10 April 2013.

80. Weil to Kosambi, 24 April and 9 May 1958, Box 9, Folder 5; 19 May 1958, Box 7, Folder 2, Papers of Damodar D. Kosambi (503), Nehru Memorial Museum and Library, New Delhi, India (hereafter “Kosambi Papers”).

81. Undated, c. 1966, Box 10, Folder 4, Kosambi Papers. The Revolutionary forebear is likely the French literary figure François Guillaume Ducray-Duminil, in whose novels secret identities were a recurring theme. See Katherine Astbury, “L’Œuvre romanesque de François Guillaume Ducray-Duminil. Par Łukasz Szkopiński,” *French Studies* 71 (2017): 418–19.

82. S. Ducray, “Normal Sequences,” *Journal of the University of Bombay* 31, 1962–3; typescript on the prime number theorem, c. 1966, both from Box 10, Folder 4, Kosambi Papers.

83. See Pannwitz to Ducray, 7 January 1965, Box 9, Folder 1, Kosambi Papers, acknowledging offprints sent to the *Zentralblatt* under Ducray’s name. *Mathematical Reviews* entries are MR0179148 (31, 3399) and MR0197433 (33, 5598). *Zentralblatt* entries are Zbl 0124.02802, Zbl 0133.29903, Zbl 0149.29301, and Zbl 0154.30004.

84. Olkar to Kosambi, 8 February 1966, Box 10, Folder 1, Kosambi Papers.

85. E.g. Erdős, 9 December 1965 and 9 February 1966; Feller, 29 February 1966, Box 10, Folder 1. Erdős, 22 August 1965, 3 and 18 November 1965, Box 10, Folder 2. Erdős, 18 April and 27 June 1965; Pitt, 1 June 1965; Feldman, 25 March 1965; MacLane, 12 March 1965, Box 11, Folder 2, Kosambi Papers. All letters to Kosambi.

86. Cf. Peter Steiner, “On the Internet, Nobody Knows You’re a Dog,” *The New Yorker*, 5 July 1993. Steiner’s famous cartoon indexed another period where changing media and norms of communication challenged routines of personification and made openings for play and transgression.

